# Shared music listening, emotional arousal and cognitive empathy enhance social presence in online interactions

**DOI:** 10.1007/s10339-026-01395-9

**Published:** 2026-10-06

**Authors:** Pyry Heikkinen, Vesa Putkinen, Mari Tervaniemi

**Affiliations:** 1 Cognitive Brain Research Unit, Centre of Excellence in Music, Mind, Body and Brain, Department of Psychology, Faculty of Medicine, University of Helsinki, PO Box 21, Haartmaninkatu 3, 00014 Helsinki, Finland; 2 Turku PET Centre, University of Turku, Turku, Finland; 3 Turku University Hospital, Turku, Finland; 4 Turku Institute for Advanced Studies, University of Turku, Turku, Finland; 5 Centre of Excellence in Music, Mind, Body, and Brain, Faculty of Educational Sciences, University of Helsinki, Helsinki, Finland

**Keywords:** Shared music listening, Social presence, Cognitive empathy, Emotional arousal, Online cooperation

## Abstract

Music can support social bonding, especially in face-to-face interactions, but its effects in online environments with reduced social cues are less understood. This study examined how shared music listening in an audio-only online setting affects social presence, affective responses, and subsequent cooperation. In a within-subjects design, 38 individuals were assigned to music-preference-matched dyads to complete cooperative tasks after listening to music in both a socially framed shared listening condition and a solo condition. Shared listening increased social presence compared with solo listening, and music-induced arousal was positively associated with social presence across conditions. Cognitive empathy moderated the effects of listening condition on both social presence and arousal, suggesting that individual differences in social-cognitive processing shape how shared listening in technology-mediated environments is experienced. However, shared music listening did not improve task performance and its effects on interaction quality were mixed. Group flow was adversely affected by shared listening, whereas dyad-level differences in music-induced arousal reduced emotional interdependence during cooperation, but only after shared listening. In addition, higher positive valence was associated with lower behavioral interdependence after shared listening but higher behavioral interdependence after solo listening. Together, these findings suggest that socially framed shared music listening can enhance the subjective social character of online interaction, while shaping interaction quality through affective alignment and perceived mutual adaptation rather than uniformly improving task-focused cooperation.

**Supplementary Information:**

The online version contains supplementary material available at https://doi.org/10.1007/s10339-026-01395-9.

## Introduction

Effective cooperation relies on a group’s ability to form, update and predict shared mental and behavioral states in ways that support common goals (Pels et al. 2018; Pels and Kleinert 2023). In face-to-face interaction, this is scaffolded by a rich set of finely coordinated social cues, including facial expressions, body language, and the precise timing of responses and turn-taking dynamics (Levinson 2016; Templeton et al. 2022). However, in online environments these cues are often reduced, delayed, or unavailable, making mutual understanding and social presence, the feeling of sharing a space and having access to the inner experience of others, more difficult to establish (Biocca et al. 2003; Gaggioli 2011; Tomprou et al. 2021). As digitally mediated interaction increasingly includes shared leisure activities with meaningful social functions, it is important to understand how social connection is formed in contexts where social cues differ from those in face-to-face interaction.

In recent years, virtual concerts, online livestreamed performances, and collaborative playlists have become common and psychologically meaningful forms of mediated musical engagement. Virtual and livestreamed concerts can support social presence, enjoyment, and feelings of connection (Kulla et al. 2024; Onderdijk et al. 2021; Swarbrick et al. 2021), while collaborative playlists enable shared engagement through music sharing, co-curation, and discovery across social and cultural contexts (Harris 2022; Park et al. 2024; Park and Kaneshiro 2022). Together, these findings indicate that shared musical experiences increasingly occur in online contexts where social functions of music may depend on cognitively constructed co-presence and shared affective experiences, in the absence of direct social cues available in physically co-present interaction.

Musical activities have long been associated with social outcomes, both by directly affecting behavior by facilitating shared attention, emotional alignment, and joint regulation of actions, as well as by supporting indirect social appraisals, such as inferring group identity and cooperative intent (Hagen and Bryant 2003; Keller et al. 2014; Mehr et al. 2020; Phillips-Silver and Keller 2012; Savage et al. 2020). For example, improvisation and musical games foster physiological and attentional alignment leading to increases in group flow (Sawyer 2006; Shehata et al. 2021). More generally, shared experiences characterized by emotional and physiological synchrony have been found to strengthen social bonds (Cheong et al. 2023).

At the same time, much of the strongest evidence for music’s social effects comes from face-to-face contexts in which motor synchronization plays a key role. Indeed, music’s prosocial effects are often contrasted with effects of interpersonal synchrony more broadly, such as tapping, marching, or rowing together (Hove and Risen 2009; Valdesolo and DeSteno 2011; Wiltermuth and Heath 2009). Although music appears to deepen these effects beyond simple rhythmic coordination (Stupacher et al. 2017), most existing paradigms do not isolate what music contributes when direct social contact and movement-based alignment are minimized. As a result, we know relatively little about whether music can support social presence in online settings where interaction is cue-reduced and sharedness must be inferred from context rather than from rich perceptual feedback and coordination.

Music preferences are especially relevant in these mediated environments, as they function as social identity cues. People form accurate perceptions of others based on their musical preferences, and music preferences are one of the most shared forms of information when people are getting to know one another (Rentfrow and Gosling 2006). This matters particularly in online environments, where identity-relevant cues may play a stronger role when direct observation of others’ behavior is limited. Indeed, prior work has shown that social identity cues can enhance social presence and support feelings of connection in computer-mediated settings (Feng et al. 2016; Schumann et al. 2017), and that when communicative cues are filtered out, people compensate by sharing more personal information or using more emotionally salient language (Antheunis et al. 2012). Shared music preferences may therefore provide a socially meaningful basis for inferring similarity, common focus, and potential emotional overlap even when partners are not visually co-present.

While much research on music’s social impact has focused on synchronization effects, the roles of social cognition and emotional activation remain less explored, despite being central to both the motivation for engaging with music and mediating our responses to it (Juslin and Västfjäll 2008). Listening to music affects social affiliation, prosocial behavior, and enhances empathy towards out-group members (Clarke et al. 2015; McDonald et al. 2022). These effects have been associated with lyrical and emotional valence of the listened music and the activation of the reward-circuitry (Curzel et al. 2024; Fukui and Toyoshima 2014; Greitemeyer 2009; Kniffin et al. 2017).

Music-induced emotions are highly sensitive to social context, with factors such as the presence of others, personal relationships, and control over music selection shaping the emotional experience (Liljeström et al. 2013). Prior studies have found that the presence of others can heighten, leave unchanged, or even dampen music-induced emotions depending on the setting and relationship between listeners (Curzel et al. 2024; Egermann et al. 2011; Sutherland et al. 2009). This makes social context especially important when considering how music may operate in online interaction.

Differences in individual characteristics add an additional level of variability to how music affects social bonding, with cognitive and affective empathy being key traits shaping social-affective experiences related to music (Miu and Vuoskoski 2017). Cognitive empathy supports the inference of others’ mental and emotional states, whereas affective empathy is more closely related to sharing or resonating with others’ feelings. These traits are relevant to music-related social experience, as empathy has been linked to both the bonding effects of music and interpersonal coordination during joint musical activity (Novembre et al. 2019; Stupacher et al. 2022). In cue-reduced environments, where people must rely more heavily on contextual cues and inference, these characteristics may play a critical role in how the social aspects of the experience are perceived.

### Current study

In the present study, we examine a specific and theoretically informative form of shared music experience: an online, auditory-only, socially framed listening episode between previously unacquainted partners. We ask if shared music listening in this social cue-reduced environment influences social and affective experiences, and whether these effects extend to subsequent cooperation during collaborative tasks. We isolate music’s affective and social-symbolic functions from richer multimodal and movement-based channels of coordination. Rather than testing fully co-present co-listening, the study asks whether a jointly initiated and socially framed listening context is sufficient to enhance social presence and cooperation when conventional interpersonal cues are limited.

Our key hypotheses are as follows: H1: Social presence will be higher in the shared listening condition, where participants listen to the same music in a jointly framed online context. H2: Stronger emotional responses to music, reflected in arousal and positive valence, will strengthen the effect of shared music listening on social presence. H3a: Cognitive empathy will enhance the effect of shared music listening on social presence. H3b: Affective empathy will magnify the association between emotional responses to music and social presence. H4a: Shared music listening will enhance cooperation. H4b: This effect will reflect increases in social presence and affective alignment. H5a: Shared music listening will enhance interaction quality. H5b: This effect will reflect increases in social presence and affective alignment.

Data, results, and discussion regarding Hypotheses H1—H3 are described in the section Social Presence and Affective Responses During Music Listening, while the section Cooperation Following Shared Music Listening focuses on H4—H5.

## Materials and methods

38 native Finnish-speaking university students (34 identifying as females, 4 as males) with an age range of 20—36 (M = 24.7, SD = 4) took part in the experiment. Participants were divided into 19 dyads (15 female-female, 1 male-male, and 3 female-male) that took part in the cooperation phase of the experiment. All participants were healthy adult volunteers, recruited via university mailing-lists and social-media sites. The amount of extracurricular musical training participants had received ranged from 0 to 15 years (M = 5.1, SD = 5.1). The highest level of education completed by participants was as follows: high school (*n* = 21), bachelor’s degree (*n* = 13), and master’s degree (*n* = 4). No other data on socio-economic status was gathered. Data collection finished in the fall of 2022. The study was approved by the University of Helsinki Research Ethics Committee in the Humanities and Social and Behavioral Sciences, and the Declaration of Helsinki was followed. The sample size was determined pragmatically based on the number of volunteers we could recruit with matching song preferences during the academic.

### Procedure

The experiment was conducted in two separate sessions. Both the prescreening session and the cooperation session were completed online, using the Zoom video-conferencing platform (version 5.8), REDCap (Harris et al. 2009, 2019) for survey data, and a custom website built for this experiment’s joint problem-solving session. During the cooperation session, participants communicated exclusively using the auditory stream, with video disabled. All participants were instructed to use a computer with an adequate internet connection for video conferencing, and to wear the headphones they would normally use when listening to music in daily life.

All candidates took part in pre-experiment screening, where they were provided information about the experiment and gave written informed consent after being verified to meet the inclusion criteria. Subsequently, participants completed the trait-level and background assessments (see Table 1 for an exhaustive list of measures used).

**Table 1:** *Overview of Measures*,* Tasks*,* and Questionnaire Subscales*

Measure/Task	Construct	Administration Phase	References
Reading the Mind in the Eyes (RMIE)	Cognitive empathy (ability to infer others’ mental states)	Pre-screening	Baron-Cohen et al. 2001
Pictorial Empathy Test (PET)	Affective empathy (emotional response to visual stimuli)	Pre-screening	Lindeman et al. 2018
Background Questionnaire	Demographic information (age, gender, education, online communication use)	Pre-screening	Custom
*Empathy Quotient-Short (EQS)*	*General empathy (exploratory; not reported)*	*Pre-screening*	Wakabayashi et al., 2006
*Short Five (S5)*	*Personality traits (exploratory; not reported)*	*Pre-screening*	Konstabel et al., 2012
*Goldsmiths Musical Sophistication Index (GOLD-MSI)*	*Musical sophistication (e.g.*,* training*,* listening habits; exploratory; not reported)*	*Pre-screening*	Müllensiefen et al., 2014
*Raven’s Progressive Matrices*	*Visuospatial problem-solving ability (exploratory; not reported)*	*Pre-screening*	Raven, 1989
Self-Assessment Manikin (SAM)	Affective responses to music (arousal, valence)	Music listening	Bradley and Lang 1994
Visual Guidance task (VG)	Cooperative problem solving (ability to give and follow instructions)	Cooperation	Wikström et al. 2021
Realistic Presented Problems task (RPP)	Divergent thinking (fluency in generating problem solutions)	Cooperation	Agnoli et al. 2016; Okuda et al. 1991
Networked Minds Social Presence measure (SPI)	Social presence	Music listening and cooperation	Harms and Biocca 2004
*SPI subscales*			
Attention Allocation	Degree of attention toward the partner	Music listening and cooperation	
Message Understanding	Clarity of communication	Cooperation	
Emotional Interdependence	Mutual affective responsiveness	Cooperation	
Affective Understanding	Ability to perceive and understand others’ emotional states	Cooperation	
Behavioral Interdependence	Coordination and synchronization of actions	Cooperation	
*Interaction-quality subscales*			
Co-flow	Group flow and shared absorption	Cooperation	Wageman et al. 2005
Psychological Safety	Interpersonal safety and willingness to take risks	Cooperation	Edmondson 1999
Social Cohesion	Sense of belonging and connectedness	Cooperation	De Kort et al. 2007; Wageman et al. 2005
Equality	Perceived fairness and balanced participation	Cooperation	Kim et al. 2017
Resonance	Degree of emotional contagion	Cooperation	based on de Waal and Preston 2017

Note. The table indicates the construct(s) assessed, the administration phase, and the reference(s) for each instrument or subscale used. Measures collected during pre-screening but not reported in the current article are italicized

Participants who completed the screening were instructed to choose 10 of their most liked songs from a list of diverse pieces provided by the authors of the study. The list was generated via the Spotify API to reflect currently popular songs from a wide range of genres and was accompanied by a playlist. Participants were instructed to choose 10 liked songs and provide a preference rating for each. Dyads were formed when at least two songs were chosen in common, ensuring a pleasant, preference-matched listening context. None of the dyad members knew each other beforehand.

**Fig. 1:**
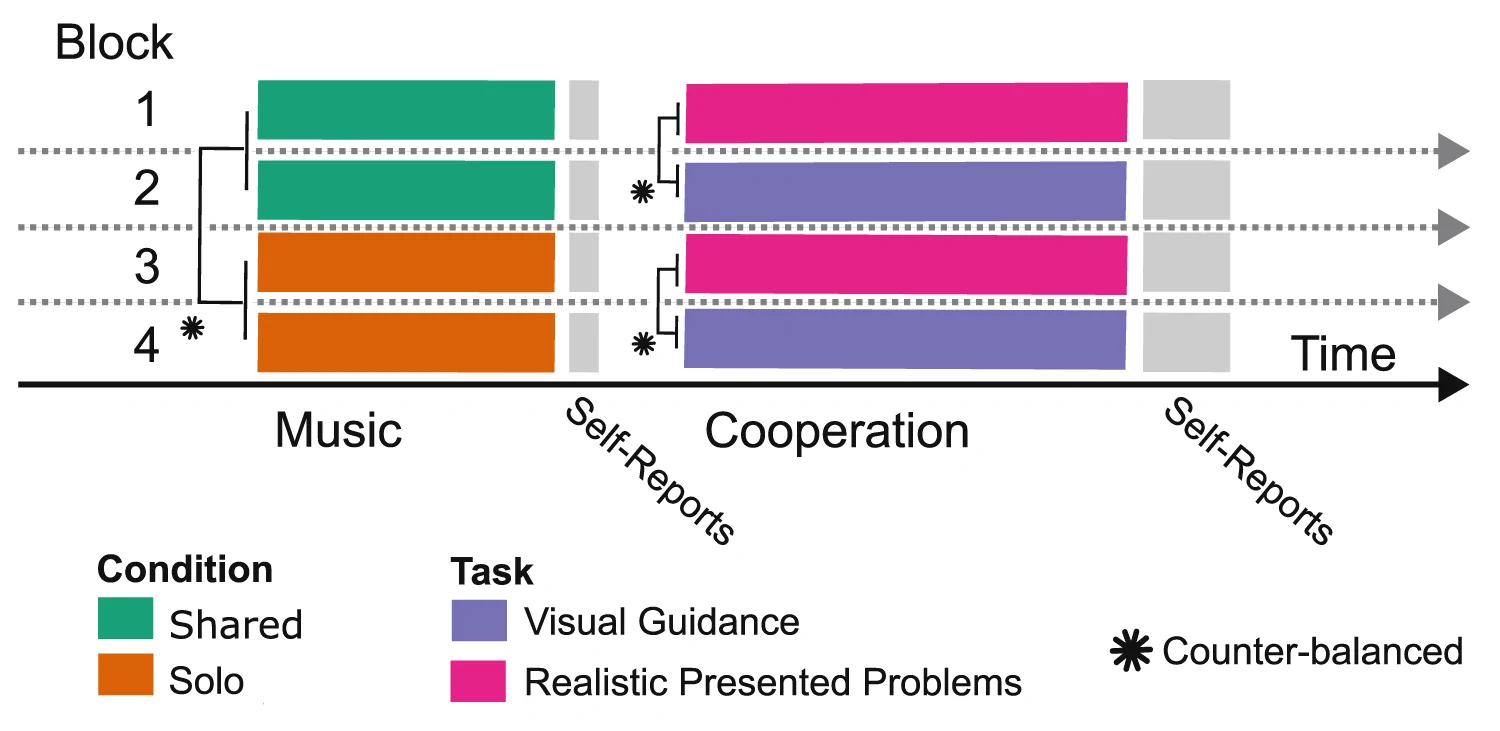
Structure of the cooperation session. The session consisted of four blocks, each including a music-listening episode, a cooperation task, and self-report measures. Listening-condition order was counterbalanced, so that both cooperation tasks were completed first in one listening condition and then in the other. Cooperation-task order was counterbalanced within listening condition

At the start of the cooperation session, participants had a chance to become familiar with each other by introducing themselves and having approximately a one-minute discussion about their latest meal. One experimental block consisted of music listening, in either a shared listening condition or a solo listening condition, followed by joint problem-solving in one of two tasks. After each song, participants completed self-reports relating to music-induced affective experiences and felt social presence during music listening. After each problem-solving task, participants completed self-reports targeting the experiential aspects of the interaction (Table 1). A full within-subjects design was used, leading to four runs per dyad, with counterbalancing of listening condition and problem-solving task (Fig. 1).

### Listening conditions

Before each music-listening episode, the experimenter provided participants with a song link and verbally introduced the listening context. In the shared listening condition, both dyad members received a link to the same song and were informed that the song was based equally on both participants’ prior song selections. The verbal instruction, given in Finnish, was: “You will now receive a link to the same song, which is based equally on the song selections made by both of you.” In the solo listening condition, dyad members received links to different songs and were informed that each song was based only on the individual participant’s own prior selections. The verbal instruction was: “You will now each receive a link to different songs. In other words, you will listen to different songs that are based only on your own individual selections.”

After both participants confirmed their readiness to begin listening, the experimenter instructed them to start the song, making sure the listening was jointly initiated. Thus, the listening manipulation combined song assignment and social framing. Participants were not informed about the condition structure beforehand. In the present study, shared listening therefore refers to a socially framed listening episode in which dyad members listened to the same song within the same online session, rather than to an interaction involving continuous interpersonal feedback during the music itself.

### Cooperation tasks

Dyads’ joint problem-solving abilities were assessed using two cooperation tasks designed to capture different aspects of cooperation. The Visual Guidance task (VG) was used to assess the participants’ ability to give and follow instructions clearly and expediently (Wikström et al. 2021). Originally designed to model a typical customer-service advisor task, the Visual Guidance task requires one participant to direct the visual search and motor actions of their partner while organizing differently shaped blocks into a grid, attempting to form a shape visible only to the advisor. Roles were alternated to balance contributions, and task complexity increased progressively. Performance in the task depends strongly on the engagement of both members and on the ability to communicate complex shapes effectively.

To assess the dyad’s ability to generate possible solutions to real-world problems, the Realistic Presented Problems task (RPP) from the Runco Creativity Assessment Battery (rCAB; used with permission), was included (Agnoli et al. 2016; Okuda et al. 1991). This task measures divergent thinking abilities, by asking participants to generate as many possible solutions as they could to three problems within a three-minute timeframe per problem. Task contribution was balanced by alternating which dyad member wrote down the solutions.

### Empathy and background measures

Trait-level measures used in the pre-screening phase consisted of assessments of cognitive and affective empathy, background information (age, gender, education level) as well as other exploratory measures, such as personality factors, but were deemed out of scope for the present publication and not formally analysed (Table 1). Cognitive empathy was measured using the Reading the Mind in the Eyes Test (RMIE; Baron-Cohen et al. 2001), and affective empathy was assessed via the Pictorial Empathy Test (PET; Lindeman et al. 2018).

The following sections present the methods, results, and related discussion in two parts. First section focuses on the effects of shared music-listening on music-induced affective responses, social presence and their relation to trait-empathy. The second section examines the effects shared music listening has on cooperation during collaborative problem-solving.

## Social presence and affective responses during music listening

This section focuses on describing the measures, statistical analysis and results relating to social presence and emotional reactions during music listening. The modulating effects of trait empathy are also examined. The findings are discussed in relation to our stated hypotheses H1—H3.

### Measures

The affective responses to music were measured with the self-assessment manikin’s arousal and valence dimensions (Bradley and Lang 1994). Social experiences during music-listening were quantified as a mean score of two questions taken from the networked minds measure of social presence (SPI): “The presence of my partner was clear to me” and “I thought about my pair during the task” (Harms and Biocca 2004).

### Statistical analysis

All analyses were conducted in R (version 4.2.2), using linear mixed-effects models (LMMs), as implemented in lme4 (Bates et al. 2015). Three separate models were built in a stepwise fashion to examine the effects of music listening on social presence, music-induced arousal and music-induced valence. In these models, all categorical variables were effect-coded (each category was compared to the overall mean). Individuals were added to the model as random intercepts. All models were assessed for normality and heteroscedasticity of residuals as well as collinearity between variables. Inclusion of the variables of interest was done in a stepwise fashion, based on the improvement of the model fit as indexed with the Akaike Information Criterion (AIC). The order of inclusion was hypothesis driven (see SI Table S1, for model comparison information for all tested models). In addition, the effect of song preference in different conditions was examined, to ensure the effects were not driven by song likability.

### Results

In general, songs in the shared condition were less preferred than ones in the solo condition, *β* = -0.18, 95% CI [-0.28, -0.07], *p* < .001. Nevertheless, songs were highly preferred in both conditions, with a mean preference rating of 8.84 (SD = 0.94) in the shared condition and 9.20 (SD = 0.85) in the solo condition. Incorporating preference into the subsequent models did not increase the fit of any of the models, so we conclude that the effect of preference does not confound our results.

#### Social presence

Social presence was best predicted by a model with fixed-effect structure of cognitive empathy × listening condition + arousal, with marginal *R*^*2*^ = 0.126 (SI Tables S2 and S3 for full fixed and random effects information, respectively).

**Fig. 2:**
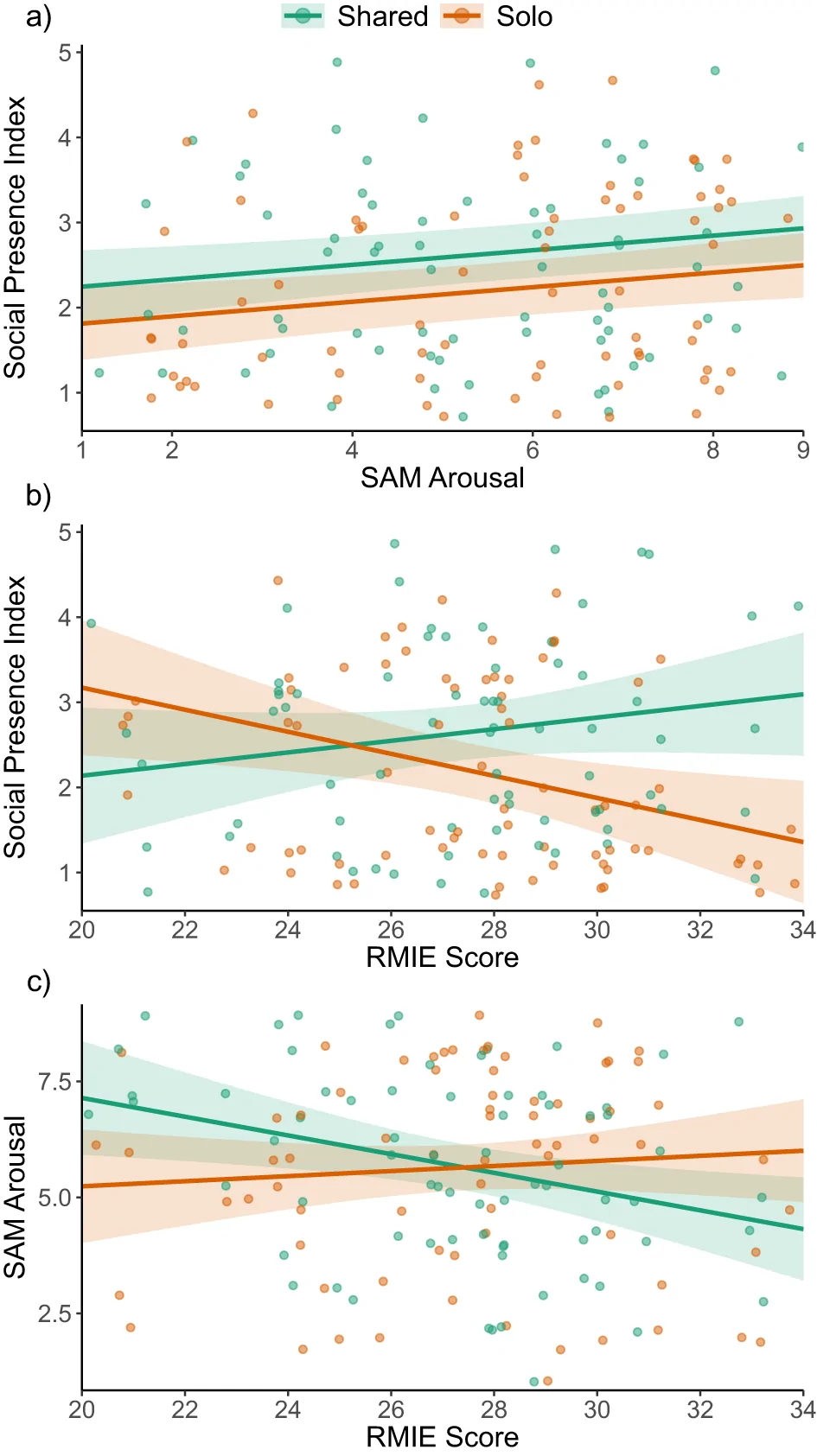
Social presence, cognitive empathy, and affective experiences during music listening. Individual data points and linear model predictions are plotted separately by listening condition. Shaded areas represent 95% confidence bands. Panel a shows the association between music-induced arousal and social presence. Panel b shows the interaction between cognitive empathy and listening condition in predicting social presence. Panel c shows the interaction between cognitive empathy and listening condition in predicting music-induced arousal

Social presence during music listening was significantly higher in the shared listening condition than in the solo listening condition, *β* = -2.50, 95% CI [-3.47, -1.52], *p* < .001. Similarly, arousal during music listening increased social presence, *β* = 0.09, 95% CI [0.02, 0.15], *p* = .006 (Fig. 2a).

Moreover, cognitive empathy (RMIE) significantly modulated the effect of the music listening condition on social presence. Higher cognitive empathy enhanced social presence in the shared condition but decreased it in the solo condition, as seen in the statistically significant interaction between cognitive empathy and listening condition, *β* = 0.10, 95% CI [0.06, 0.13], *p* < .001 (Fig. 2b).

#### Affective reactions

Music-induced arousal was best predicted by a model with a fixed-effect structure of listening condition × cognitive empathy, with marginal *R*^*2*^ = 0.049 (SI Tables S2 and S3).

The model revealed a significant main effect of listening condition on felt arousal, *β* = 3.52, 95% CI [0.68, 6.36], *p* = .015, which was modulated by cognitive empathy: higher cognitive empathy scores were associated with decreased arousal in the shared condition but not in the solo condition, *β* = -0.13, 95% CI [-0.23, -0.03], *p* = .015 (Fig. 2c).

The fit of the model explaining valence during music listening did not improve with the addition of any variables of interest, so we conclude there are no meaningful effects to be reported (SI Tables S2 and S3).

### Discussion: social presence and affective responses during music listening

In accordance with our hypothesis (H1), socially framed shared music listening increased social presence compared with solo listening. This indicates that even in an online setting with limited interpersonal cues, a shared listening episode can make the interaction partner more salient during the musical experience. Importantly, the shared condition did not provide continuous expressive feedback from the partner during listening. The effect therefore appears to reflect the social nature of the listening context itself: participants knew that the music was shared, simultaneously initiated, and based on overlapping musical preferences. This is in line with previous work showing that music preference can be used as an accurate source of identity information and this information can, in turn, enhance social presence (Feng et al. 2016; Rentfrow and Gosling 2006; Schumann et al. 2017).

H2 was partially supported. Music-induced arousal was connected to social presence, whereas no effect of valence was observed. Interestingly, the arousal effect was present irrespective of whether the listening situation was socially framed or not, indicating that the effect of music was more general than anticipated. This may reflect the broader social salience of the experimental context itself. Even in the solo listening condition, participants were aware that they were taking part in a dyadic study and would subsequently interact with one another. Nevertheless, the results underscore arousal as the critical affective component, suggesting that the intensity of the emotion experienced, rather than its tone, is more influential when inferring the social nature of the experience.

The finding that cognitive empathy moderates the effect of listening condition on social presence offers partial support for H3. Participants with higher cognitive empathy showed a stronger increase in social presence in the shared condition, compared to the solo condition. This shows that dispositional traits in social cognitive processing are particularly relevant for social presence when the shared experience does not include direct social cues, but needs to be inferred from contextual information. It is also in accordance with research linking empathic perspective taking to coordination and social bonding in face-to-face musical contexts (Novembre et al. 2019; Stupacher et al. 2022).

As the listening phase provided socially framed sharedness rather than continuous interpersonal feedback, the finding that higher cognitive empathy was associated with lower arousal in the shared condition allows several plausible interpretations. Higher cognitive empathy may have supported a more reflective orientation to the shared situation, a more regulated affective response, or a shift from music-centered to more interaction-concerned cognitive orientation. Even so, this indicates that social presence and arousal were not simply interchangeable aspects of the experience, reflecting connected, but distinct facets of the experience, distinguishable by their relation to social-cognitive processes.

By contrast, affective empathy did not significantly moderate these effects. In a cue-reduced online setting without access to a partner’s ongoing expressive cues, this pattern is consistent with the view that affective empathy—typically engaged through direct perception of others’ emotional expressions—plays a more limited role when such input is unavailable (de Waal and Preston 2017). This supports the conclusion that, in socially framed shared listening under cue-reduced conditions, experienced social presence depends more on inferred sharedness and partner salience than on direct affective resonance.

## Cooperation following shared music listening

This section focuses on describing the measures, statistical analysis, and results relating to cooperative task performance and interaction quality following shared music listening, as well as the modulation by trait empathy. The findings are discussed in relation to our stated hypotheses H4 and H5.

### Measures

Task performance was quantified in the Visual Guidance task (VG) as the number of successfully finished puzzles within a 9-minute timeframe. In the Realistic Presented Problems task (RPP), performance is quantified by fluency of production, i.e. the number of responses given in 9 min. The results of both tasks were converted to Z-scores to make the scores comparable, and to eliminate the effect of different measurement units in the statistical models (Andrade 2021).

To examine the interaction quality, networked minds for social presence questionnaire (SPI) was used, together with a tailored set of questions aiming to measure relevant dimensions of successful interpersonal interaction identified from previous literature (see Table 1; De Kort et al. 2007; de Waal and Preston 2017; Edmondson 1999; Kim et al. 2017; Wageman et al. 2005). As the participants were not allowed to see each other, two questions relating to behavioral resonance focused on language (mirroring of tone and word choices).

### Statistical analysis

#### Questionnaire dimension reduction

To reduce the number of dimensions in the self-reports on interaction quality in further analysis, the 11 self-report questionnaire subscales in the cooperation questionnaires were run through principal component analysis in R, utilizing built-in functions and the Factoextra package (Kassambara and Mundt 2020), with the initial values zero centered and scaled to have unit variance.

**Fig. 3:**
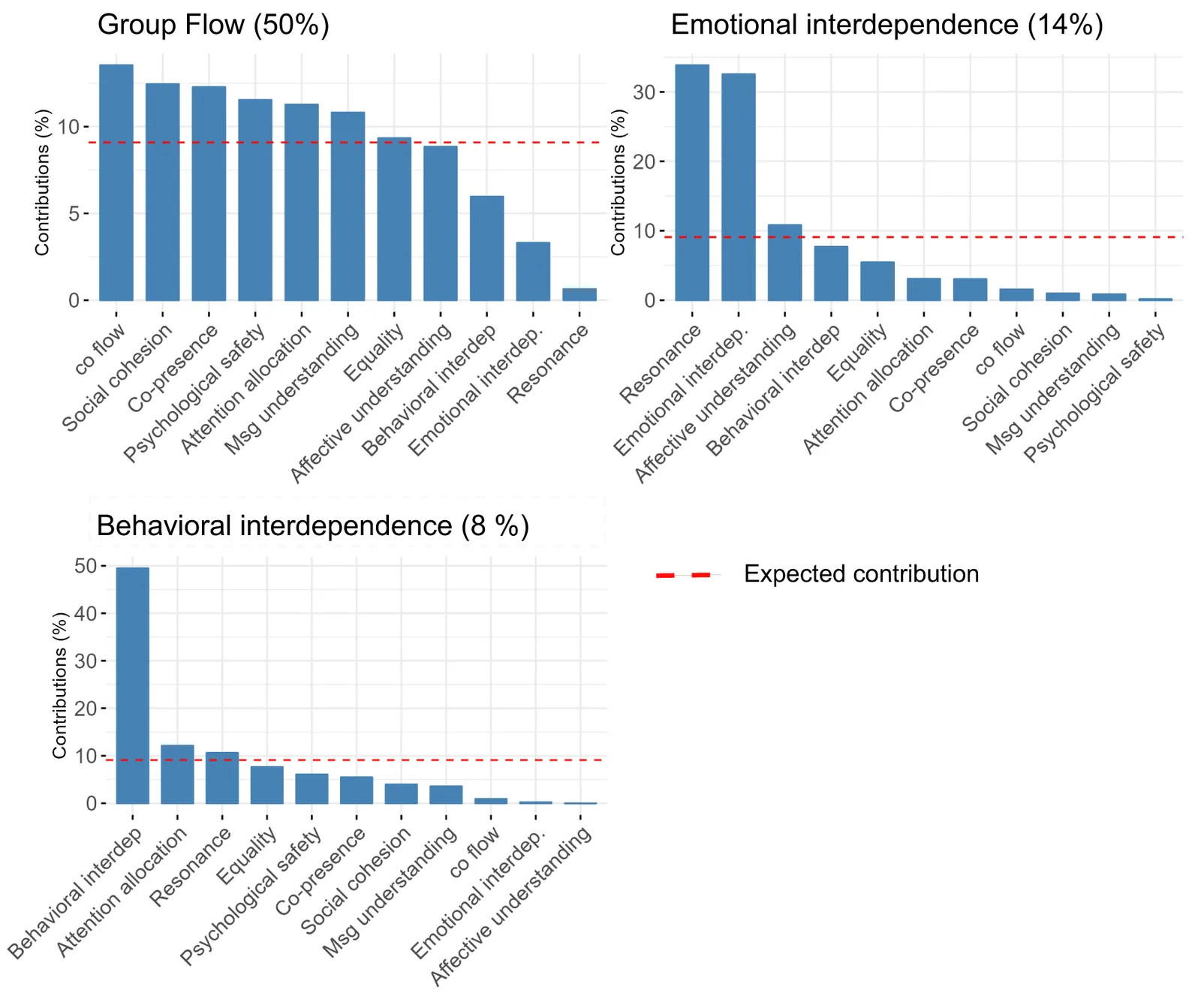
Relative contribution of questionnaire subscales to the retained principal components. Factor loadings are shown for the three components retained for further analysis, with their percentage of variance explained in brackets. The dotted red line denotes the expected contribution percentage if contributions were evenly distributed across subscales

Three components were selected for further analysis based on explained variance and the interpretability of their loading patterns (Fig. 3). Together, these components accounted for approximately 72% of the variance (SI Table S4). We refer to them as Group Flow (PC1), Emotional Interdependence (PC2), and Behavioral Interdependence (PC3). These labels reflect our interpretation of the dominant contributing subscales and do not correspond directly to the original questionnaire scales. Emotional and behavioral interdependence refer to the perceived reciprocity and felt connection between dyad members’ affective experiences and performed actions, respectively.

#### Statistical modelling of task performance and interaction quality

The effect of the chosen principal components on task performance was examined with a linear mixed-effects model, with the dyad mean of each component as an independent variable, and dyad as a random intercept. To examine whether music listening affected subsequent cooperation, separate linear mixed-effects models were built for four outcome measures: task performance, expressed as a Z-score, and the three interaction-quality components described above. Interaction-quality data from the Visual Guidance task were excluded for one dyad because of missing questionnaire answers.

The independent variables of interest were the effect of music listening condition (shared vs. solo) and music-induced affective experiences. To examine whether affective responses during music listening were related to subsequent cooperation at the dyad level, we calculated both the dyad mean and the absolute dyad difference for music-induced arousal and valence. Dyad means index the overall affective tone or intensity of the listening episode within the pair. Dyad difference indexes affective alignment, defined here as the degree to which partners showed similar affective responses to the listening episode. This approach follows work suggesting that shared experiences are partly characterized by affective alignment between individuals, and that such alignment is relevant for social connection and subsequent interpersonal coordination (Cheong et al. 2023; Li et al. 2021).

The model building procedure was conducted for all the dependent variables separately, following the same stepwise procedure as with music listening (SI Tables S5 – S8 for model selection results for each outcome factor). In the models concerned with the quality of the interaction, the contribution of task performance was also included in the analysis as an independent variable to control for the effect overall performance might have. Task repetition (first vs. second time performing the task), task type (RPP vs. VG) and their interaction were included in all the models as fixed effects to control for their effects.

### Results

#### Task performance

**Fig. 4:**
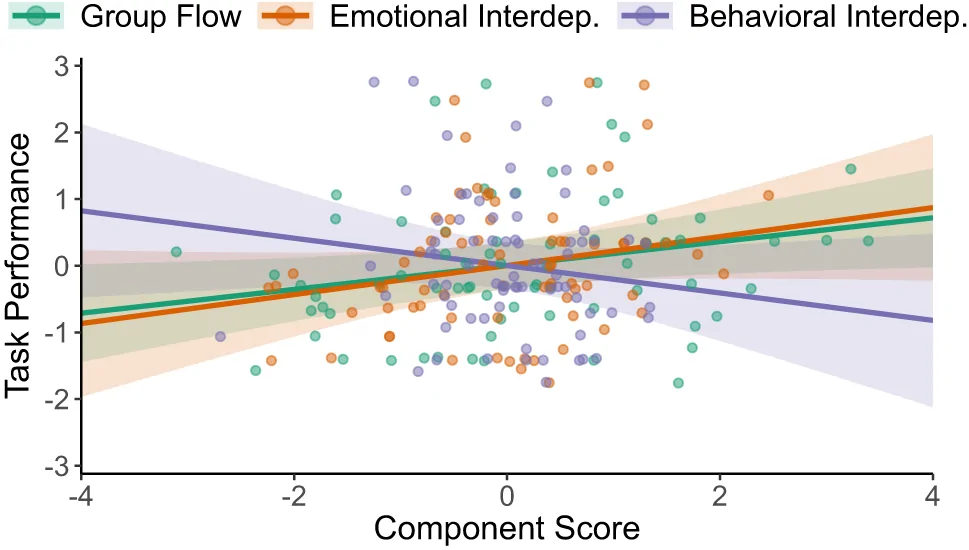
Relationships between task performance and principal component scores. Individual data points and linear model predictions are shown with 95% confidence bands for the relationship between cooperation-task performance and each retained principal component. Group Flow (PC1) was significantly associated with task performance

Task performance increased with group flow, *β* = 0.18, 95% CI [0.02, 0.34], *p* = .03, while emotional interdependence, *β* = 0.22, 95% CI [-0.04, 0.48], *p* = .10, and behavioral interdependence, *β* = -0.21, 95% CI [-0.52, 0.11], *p* = .195, were not significantly related to task performance (Fig. 4). Marginal *R*^2^ for the model was 0.156. None of the music-related variables of interest improved the model fit and thus are not reported (SI Table S5).

#### Group flow

Model best explaining group flow during cooperation had a fixed-effect structure of task repetition × task + task performance + listening condition, with Marginal *R*² of 0.112 (see SI Tables S9 and S10 for full fixed effects and model information of all the models, respectively).

**Fig. 5:**
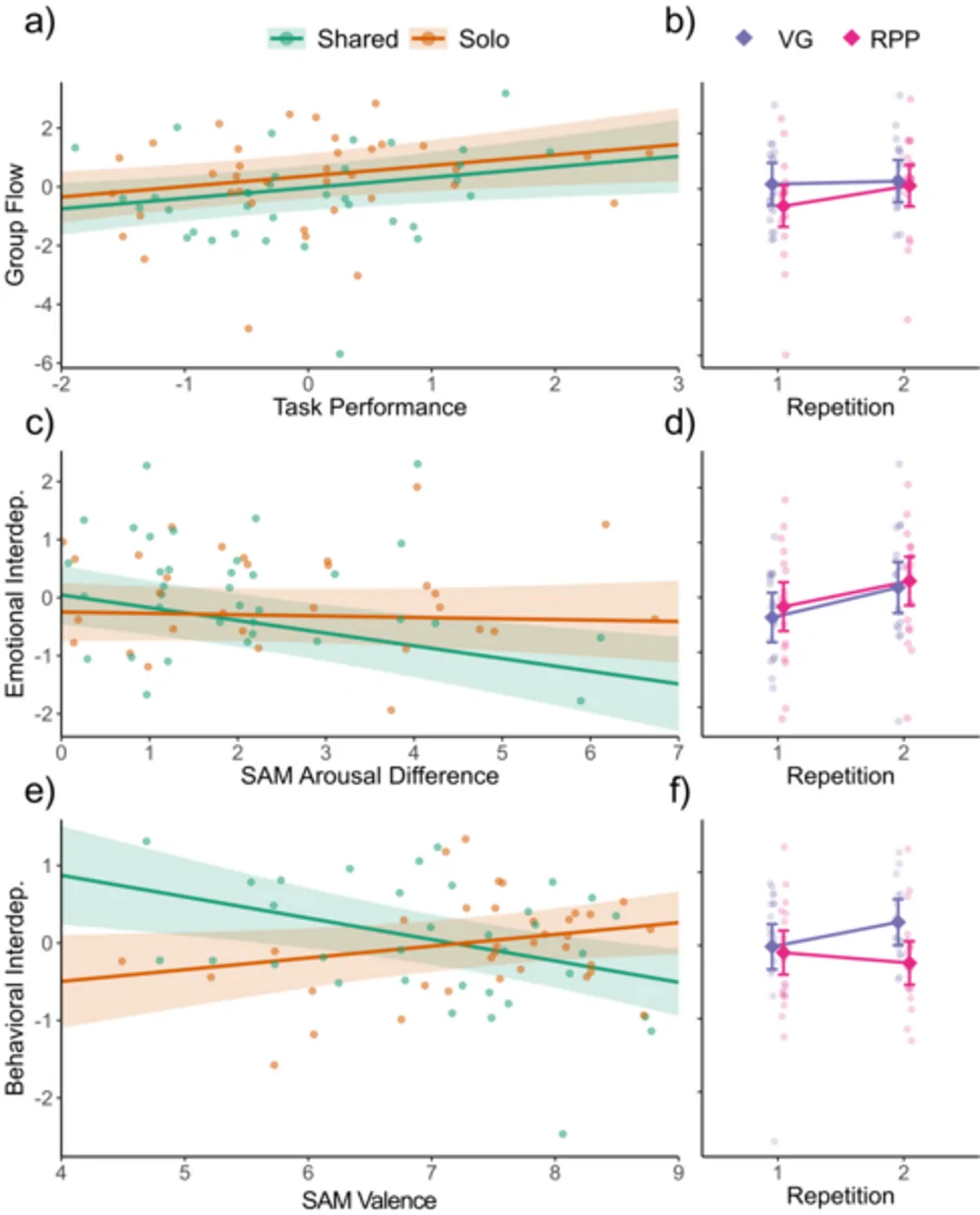
Predictors of interaction quality during cooperation. Individual data points and model-based summaries are shown for the main predictors of interaction quality during cooperation. In the left column, lines show fitted model predictions plotted separately by listening condition, and shaded areas represent 95% confidence bands. Panel a shows Group Flow as a function of task performance and listening condition. Panel c shows Emotional Interdependence as a function of dyad-level music-induced arousal difference and listening condition. Panel e shows Behavioral Interdependence as a function of dyad mean music-induced valence and listening condition. In the right column, points and error bars show estimated marginal means and 95% confidence intervals for task repetition by task type. Panel c shows these adjusted means for Group Flow, Panel d for Emotional Interdependence, and Panel f for Behavioral Interdependence. VG = Visual Guidance task; RPP = Realistic Presented Problems task

Group flow during the cooperation tasks was significantly lower after the shared music listening condition, and higher after the solo listening condition, *β* = -0.20, 95% CI [-0.38, -0.02], *p* = .03, while task performance was positively associated with group flow, *β* = 0.36, 95% CI [0.07, 0.64], *p* = .015 (Fig. 5a). Task repetition increased group flow, *β* = -0.21, 95% CI [-0.41, -0.01], *p* = .037, and there was less group flow in the Realistic Presented Problems task, compared to the Visual Guidance task, *β* = -0.24, 95% CI [-0.42, -0.06], *p* = .011 (Fig. 5b).

#### Emotional interdependence

Emotional interdependence was best explained by a fixed-effects model structure of task repetition × task + arousal difference + listening condition × arousal difference, with marginal *R*² of 0.127 (SI Tables S9 and S10).

After shared music listening, greater arousal difference between dyad members predicted lower emotional interdependence, *β* = -0.10, 95% CI [-0.18, -0.01], *p* = .026 (Fig. 5c). Similarly to group flow, emotional interdependence was also increased by task repetition, *β* = -0.25, 95% CI [-0.38, -0.13], *p* < .001 (Fig. 5d).

#### Behavioral interdependence

Behavioral interdependence was best explained by a model with a fixed-effects structure of repetition × task + task performance + mean valence × listening condition, with a marginal *R*^2^ of 0.256 (SI Tables S9 and S10).

Behavioral interdependence was predicted by an interaction between mean valence during music listening and the listening condition, *β* = -0.21, 95% CI [-0.32, -0.11], *p* < .001 (Fig. 5e). In the shared music listening condition, higher valence was associated with reduced behavioral interdependence during cooperation, while in the solo condition, higher valence was associated with increased behavioral interdependence. Listening condition showed also a main effect, *β* = 1.54, 95% CI [0.75, 2.33], *p* < .001.

Compared to the RPP task, a stronger increase in behavioral interdependence between task repetitions was observed in the VG task, as seen in the task by repetition interaction, *β* = 0.12, 95% CI [0.01, 0.23], *p* = .036 (Fig. 5f). Repetition showed no significant effect, *β* = -0.05, 95% CI [-0.17, 0.07], *p* = .43, whereas behavioral interdependence was lower in the realistic presented problems task, compared to the visual guidance task, *β* = -0.16, 95% CI [-0.27, -0.05], *p* = .005. Finally, higher behavioral interdependence was also explained by lower task performance, *β* = -0.22, 95% CI [-0.38, -0.06], *p* = .006.

### Discussion: cooperation following shared music listening

Contrary to H4, shared music listening did not enhance task-oriented cooperation performance, as none of the music-related variables improved the task performance model. Thus, the increase in social presence observed during the listening phase did not lead to more successful performance on the subsequent cooperative tasks. Here, cooperation happened within a controlled environment requiring goal-directed communication, role coordination, and task-specific focus. Within this rather rigid structure, it is possible that other contextual factors superseded more subtle effects music listening could have had if participation in the interaction was more freely initiated and maintained.

The findings for interaction quality provided a more differentiated picture. In contrast to H5, group flow was lower after shared listening than after solo listening, even though group flow was positively associated with task performance. This suggests that shared listening did not uniformly facilitate the subjective quality of subsequent cooperation. The socially framed listening context may have increased partner salience and affective involvement, but these qualities did not directly translate into the task-focused coordination reflected in group flow.

Greater dyad-level difference in music-induced arousal predicted lower emotional interdependence during subsequent cooperation, but specifically after shared listening. Thus, the relevant affective predictor was not simply the overall arousal level of the dyad, but whether the shared episode elicited comparable levels of affective activation in both partners. This pattern is consistent with work showing that shared experiences support social connection through affective alignment and synchronized affect (Cheong et al. 2023), and that shared emotional states can shape later dyadic coordination and responsiveness (Bieńkiewicz et al. 2021; Li et al. 2021). In the shared condition, arousal similarity indexed similarity in responses to a common event, whereas in the solo condition, it reflected overall arousal due to music. Hence, one possibility is that the knowledge of sharing the same musical episode made participants’ affective states more relevant to the subsequent interaction, so that similar arousal responses supported a more comparable orientation entering the cooperation task. A complementary possibility is that arousal similarity reflected a broader dyadic tendency to have more similar affective responses in a given situation, which may also have shaped how emotionally reciprocal interaction during cooperation was experienced as.

Also, in contrast with H5, higher positive valence during shared listening was associated with lower behavioral interdependence during subsequent cooperation, whereas an opposite association was observed after solo listening. This is noteworthy because positive affect is generally expected to support social interaction, cooperation, and perceived task functioning, partly through group resources such as cohesion, coordination, and teamwork (Barsade 2002; Knight and Eisenkraft 2015; Peñalver et al. 2019). One possibility is that positive valence is carried into the cooperation phase differently across listening contexts. After solo listening, it may have promoted openness and readiness to engage with the partner. After shared listening, however, positive valence was embedded in an already socially framed episode and may have reduced the need for further perceived mutual adaptation during the task. Moreover, behavioral interdependence should not be equated with better cooperation. The component reflected perceived mutual adaptation, whereas the tasks also required role differentiation, clear instruction-giving, and efficient division of labor. Effective coordination in such tasks may depend on complementarity rather than stronger alignment per se (Chidichimo et al. 2026; Wallot et al. 2016). Consistent with this interpretation, the association between behavioral interdependence and task performance was negative in both relevant models. Although behavioral interdependence did not significantly predict task performance, higher behavioral interdependence was significantly explained by lower task performance in the model that also included mean valence, listening condition, and their interaction as music-related predictors.

## General discussion

The present study examined whether socially framed shared music listening affects social presence and subsequent cooperation in an online audio-only setting. Using a within-subject design, we found that shared listening increased social presence even when continuous auditory, visual, and motor expressive cues were unavailable. This is notable because many accounts of music’s social effects emphasize embodied coordination, interpersonal synchrony, and motor alignment as central mechanisms of social bonding (Mehr et al. 2020; Savage et al. 2020). The present study isolated a more minimal form of shared musical experience: a jointly initiated and socially framed listening episode without continuous interpersonal feedback during the music itself. The findings therefore suggest that music can contribute to social experience in online interaction not only through overt synchronization or co-present actions, but also through contextual cues of sharedness, affective engagement, as well as preference-based social meanings and identity inference.

This finding is consistent with theories of mediated social presence, according to which the sense of another person’s psychological availability depends on the cues available in the interaction context (Biocca et al. 2003; Gaggioli 2011). In face-to-face interaction, social presence is supported by a rich set of expressive, bodily, and temporal cues, including facial expressions, gesture, posture, and response timing (Levinson 2016; Templeton et al. 2022). In online environments, these cues are often reduced or altered, requiring participants to rely more strongly on contextual, symbolic, or identity-relevant information (Schumann et al. 2017; Tomprou et al. 2021). In the present study, participants did not receive ongoing expressive feedback from their partner during listening, but they knew that the listening episode was shared, jointly initiated, and based on overlapping musical preferences. This appears to have been sufficient to make the partner more salient during the listening experience.

Music may be especially suited to this kind of socially framed inference because it carries affective, personal, and identity-relevant meanings and is used a cue for similarity, personality, and group membership (Rentfrow and Gosling 2006). Shared musical preference may therefore provide a basis for inferring common focus, similarity, and potential emotional overlap, even in the absence of visual co-presence. In this sense, the social effect of shared listening in the present study arose from the combination of music’s affective qualities and its role as a meaningful social cue.

The affective arousal increasing social presence further suggests that the intensity of the musical response, rather than positive valence, was most relevant for social presence. The broader pattern also indicates that social presence was not reducible to affective activation, as cognitive empathy shaped the shared-listening effect in a partly distinct way. This interpretation is also consistent with the absence of a corresponding affective empathy effect, as the listening phase offered no direct access to the partner’s expressive emotional cues.

The findings highlight the importance of individual social-cognitive processing in cue-reduced musical interaction. In the present study, the social character of the listening situation had to be inferred from contextual information rather than perceived through ongoing interpersonal feedback. The observed role of cognitive empathy is consistent with previous work linking empathy to music-related social experience and interpersonal coordination (Miu and Vuoskoski 2017; Stupacher et al. 2022; Vuoskoski et al. 2016). More broadly, this suggests that shared listening in online contexts depends not only on the musical stimulus itself, but also on the listener’s sensitivity to the partner-relevant meaning of the situation. The social effects of music may therefore be strongest when affective engagement, contextual sharedness, and social-cognitive sensitivity converge.

At the same time, the cooperation findings qualify a simple social-enhancement account of shared music listening. Although shared listening increased social presence during the listening phase, this increase was not accompanied by improved task performance. This dissociation suggests that social presence and task-oriented cooperation are related but separable aspects of interaction. Previous studies have linked music to affiliation, empathy, prosocial behavior, and cooperation (Clarke et al. 2015; Greitemeyer 2009; Kniffin et al. 2017; McDonald et al. 2022). However, these outcomes do not necessarily depend on the same processes as task-focused collaborative performance. The present findings suggest that music-related social presence may be more closely connected to felt co-presence and partner salience than to immediate improvements in cooperative output.

This distinction is particularly relevant in online interaction, where the cues that support social connection may differ from those required for efficient collaboration. The cooperative tasks in the present study required goal-directed communication, role coordination, and task-specific abilities. By contrast, the shared listening episode primarily involved affective and socially framed co-experience. Group flow, associated with better task performance, depends on coordinated attention, shared goals, reciprocal responsiveness, and task-relevant mutual regulation (Pels et al. 2018; Pels and Kleinert 2023). The interpersonal orientation supported by shared listening therefore did not directly map onto the demands of subsequent goal-directed cooperation. The lower group flow following shared listening further suggests that heightened social presence did not necessarily support the subjective fluency of subsequent task-focused interaction.

The interaction-quality findings nevertheless indicate that shared listening was relevant for later cooperation at the level of subjective interpersonal experience. Affective alignment provides one possible bridge between shared listening and subsequent interaction quality. Shared experiences can support social connection through emotional, physiological, motor, and cognitive alignment, with synchronized affect playing an important role in social bonding (Cheong et al. 2023). Shared emotional states can also facilitate later dyadic coordination, suggesting that affective commonality before an interaction may shape how responsive and mutually attuned the subsequent exchange feels (Li et al. 2021). More broadly, emotion and joint action are closely intertwined, such that affective states influence how people attend to, interpret, and coordinate with others during interaction (Bieńkiewicz et al. 2021). In the present study, affective alignment appeared most relevant to the subjective emotional quality of cooperation rather than to objective task performance. This may be especially relevant after shared listening, where affective similarity reflects responses to a common musical event rather than parallel responses to separate individual experiences.

The findings concerning behavioral interdependence further suggest that positive affect does not exert uniform effects across all forms of cooperation. Positive valence was associated with greater behavioral interdependence after solo listening, but with lower behavioral interdependence after shared listening. This partly diverges from work linking positive affect and emotional contagion to cooperation, social integration, and perceived task functioning partly through group resources such as cohesion, coordination, and teamwork (Barsade 2002; Knight and Eisenkraft 2015; Peñalver et al. 2019). In the present study, positive affect may have carried into the cooperation phase differently across listening contexts. After solo listening, it may have promoted openness and readiness to engage with the partner. After shared listening, however, positive affect was embedded in an already socially framed episode and may have reduced the perceived need for further mutual adaptation during the task.

This interpretation is also consistent with the broader distinction between behavioral interdependence and task performance. Behavioral interdependence reflected perceived mutual adaptation, whereas the tasks also required role differentiation, clear instruction-giving, and efficient division of labor. Effective coordination in such tasks may depend on complementarity rather than stronger alignment per se (Chidichimo et al. 2026; Wallot et al. 2016). Thus, lower behavioral interdependence after positively experienced shared listening does not necessarily reflect worse cooperation. Instead, it may reflect that perceived mutual adaptation was less central to the way participants experienced the subsequent task. This interpretation warrants further research and should be considered cautiously. Nevertheless, it is consistent with the negative association between behavioral interdependence and task performance, when music-related factors were included in the model.

Taken together, the results suggest that shared music listening shapes different layers of interpersonal experience in different ways. It increased perceived social presence, while its links to subsequent cooperation were limited to specific aspects of subjective interaction quality and did not extend to objective task performance. This pattern is theoretically important because it argues against treating the social effects of music as a single general enhancement of interaction. Instead, shared music appears to influence online interaction through multiple partially separable pathways, including partner salience, affective engagement, social-cognitive inference, emotional reciprocity, and perceived behavioral adaptation.

These findings have implications for digitally mediated musical engagement. Online concerts, livestreamed performances, collaborative playlists, and other forms of mediated music participation have become increasingly common contexts for social connection (Harris 2022; Onderdijk et al. 2021; Swarbrick et al. 2021; Upham et al. 2024). The present study suggests that even a minimal, socially framed listening episode can enhance social presence in an auditory-only setting. At the same time, the cooperation findings indicate that the social value of shared music may be most robust for perceived co-presence and affective connection, whereas benefits for instrumental collaboration likely depend on the fit between the musical experience, the subsequent task, and the form of coordination required.

As the study did not include a non-musical shared-content condition, the findings are best understood as effects of socially framed shared music listening rather than as evidence for music-specific mechanisms. Similar effects may occur in other jointly framed, emotionally engaging, or culturally meaningful online activities. Still, music remains theoretically relevant because it combines affective engagement, preference-based identity information, temporal structure, and social-symbolic meaning into a particularly salient and widely practiced form of social interaction.

### Limitations and future directions

While these findings provide new insight into shared music listening in online social interaction, they should be interpreted within several constraints. First, the sample was relatively small and homogeneous, consisting of Finnish university students, most of whom identified as female. This limits the generalizability of the findings across age, cultural background, musical background, and gender. Given the gender imbalance and sample size, the present study could not evaluate whether gender-related differences or gender-specificity in auditory emotion recognition or social-affective processing shaped affective responses to music, social presence, or subsequent cooperation. Second, the data were collected while the COVID-19 pandemic was still affecting everyday social interaction and increasing reliance on mediated communication. Participants’ familiarity with, fatigue from, or attitudes toward online interaction may therefore have differed from those in other periods. The practical implications of the present findings for online interaction should therefore be considered with some caution.

Third, the audio-only interaction model allowed us to focus on music-induced affective responses and the role of social cognition when visual and bodily social cues were minimized. However, this setting does not capture the full richness of many online interactions, which often include video, text, emojis, shared interfaces, or other forms of multimodal feedback. Finally, because the shared listening condition was explicitly socially framed, the increase in social presence may partly reflect participants’ response to the experimental framing rather than effects of the musical experience alone. At the same time, this framing was central to the present research question. The findings should therefore be interpreted as evidence of the social effects of a jointly initiated and socially framed listening context, rather than as evidence for music listening independent of contextual framing.

Future research should address these constraints by studying more diverse samples, comparing audio-only interaction with richer multimodal online settings, and including non-musical shared control conditions. It would also be useful to examine how specific musical features, such as tempo, genre, familiarity, preference, and emotional intensity, shape social presence and cooperation in mediated interaction.

## Conclusion

In conclusion, socially framed shared music listening increased social presence in an online auditory-only interaction, particularly in relation to music-induced arousal and cognitive empathy. However, this enhanced social presence did not translate into improved task performance, and the effects on interaction quality varied across affective and cooperative dimensions. The findings therefore suggest that shared music can support the subjective social character of mediated interaction, while its consequences for task-oriented cooperation depend on affective alignment, social framing, and the specific interpersonal demands of the task.

## Supplementary Information

Below is the link to the electronic supplementary material.

Supplementary Material 1

## Data Availability

Analysis scripts and variables used are publicly available on OSF at OSF.IO/NXACP. This study was not preregistered.

## References

[CR1] Agnoli S, Corazza GE, Runco MA (2016) Estimating Creativity with a Multiple-Measurement Approach Within Scientific and Artistic Domains. Creativity Res J 28(2):171–176. 10.1080/10400419.2016.1162475

[CR2] Andrade C (2021) Z Scores, Standard Scores, and Composite Test Scores Explained. Indian J Psychol Med 43(6):555–557. 10.1177/02537176211046525 PMC8826187 35210687

[CR3] Antheunis ML, Schouten AP, Valkenburg PM, Peter J (2012) Interactive Uncertainty Reduction Strategies and Verbal Affection in Computer-Mediated Communication. Communication Res 39(6):757–780. 10.1177/0093650211410420

[CR4] Baron-Cohen S, Wheelwright S, Hill J, Raste Y, Plumb I (2001) The Reading the Mind in the Eyes Test revised version: A study with normal adults, and adults with Asperger syndrome or high-functioning autism. J Child Psychol Psychiatry Allied Discip 42(2):241–251 11280420

[CR5] Barsade SG (2002) The Ripple Effect: Emotional Contagion and its Influence on Group Behavior. Adm Sci Q 47(4):644–675. 10.2307/3094912

[CR6] Bates D, Mächler M, Bolker B, Walker S (2015) Fitting Linear Mixed-Effects Models Using lme4. J Stat Softw 67:1–48. 10.18637/jss.v067.i01

[CR7] Bieńkiewicz MMN, Smykovskyi AP, Olugbade T, Janaqi S, Camurri A, Bianchi-Berthouze N, Björkman M, Bardy BG (2021) Bridging the gap between emotion and joint action. Neurosci Biobehavioral Reviews 131:806–833. 10.1016/j.neubiorev.2021.08.014 34418437

[CR8] Biocca F, Harms C, Burgoon JK (2003) Toward a more robust theory and measure of social presence: Review and suggested criteria. Presence: Teleoperators Virtual Environ 12(5):456–480. 10.1162/105474603322761270

[CR9] Bradley MM, Lang PJ (1994) Measuring emotion: The self-assessment manikin and the semantic differential. J Behav Ther Exp Psychiatry 25(1):49–59. 10.1016/0005-7916(94)90063-9 7962581

[CR10] Cheong JH, Molani Z, Sadhukha S, Chang LJ (2023) Synchronized affect in shared experiences strengthens social connection. Commun Biology 6(1):1–14. 10.1038/s42003-023-05461-2 PMC10613250 37898664

[CR11] Chidichimo E, Luppi AI, Mediano PAM, Leong V, Dumas G, Canales-Johnson A, Bethlehem RAI (2026) Towards an informational account of interpersonal coordination. Nat Rev Neurosci 27(2):121–137. 10.1038/s41583-025-00989-0 41249627

[CR12] Clarke E, DeNora T, Vuoskoski J (2015) Music, empathy and cultural understanding. Phys Life Rev 15:61–88. 10.1016/j.plrev.2015.09.001 26419700

[CR13] Curzel F, Osiurak F, Trân E, Tillmann B, Ripollés P, Ferreri L (2024) Enhancing musical pleasure through shared musical experience. iScience 27(6):109964. 10.1016/j.isci.2024.109964 PMC11145343 38832017

[CR15] de Waal FBM, Preston SD (2017) Mammalian empathy: Behavioural manifestations and neural basis. Nat Rev Neurosci 18(8):498–509. 10.1038/nrn.2017.72 28655877

[CR16] Edmondson A (1999) Psychological Safety and Learning Behavior in Work Teams. Adm Sci Q 44(2):350–383. 10.2307/2666999

[CR17] Egermann H, Sutherland ME, Grewe O, Nagel F, Kopiez R, Altenmüller E (2011) Does music listening in a social context alter experience? A physiological and psychological perspective on emotion. Musicae Sci 15(3):307–323. 10.1177/1029864911399497

[CR18] Feng B, Li S, Li N (2016) Is a Profile Worth a Thousand Words? How Online Support-Seeker’s Profile Features May Influence the Quality of Received Support Messages. Communication Res 43(2):253–276. 10.1177/0093650213510942

[CR19] Fukui H, Toyoshima K (2014) Chill-inducing music enhances altruism in humans. Front Psychol. 10.3389/fpsyg.2014.01215 PMC4211412 25389411

[CR20] Gaggioli A (2011) Networked Flow: A Framework for Understanding the Dynamics of Creative Collaboration in Educational and Training Settings. Open Educ J 4(1):41–49. 10.2174/1874920801104010041

[CR21] Greitemeyer T (2009) Effects of songs with prosocial lyrics on prosocial thoughts, affect, and behavior. J Exp Soc Psychol 45(1):186–190. 10.1016/j.jesp.2008.08.003 19648562

[CR22] Hagen EH, Bryant GA (2003) Music and dance as a coalition signaling system. Hum Nat 14(1):21–51. 10.1007/s12110-003-1015-z 26189987

[CR23] Harms C, Biocca F (2004) *Internal Consistency and Reliability of the Networked Minds Measure of Social Presence*. 7

[CR24] Harris I (2022) Collaborative Playlist-Making: Musical Interaction via Digitally Mediated Co-Curation. Music Sci 5:20592043221076956. 10.1177/20592043221076956

[CR26] Harris PA, Taylor R, Thielke R, Payne J, Gonzalez N, Conde JG (2009) Research electronic data capture (REDCap)—A metadata-driven methodology and workflow process for providing translational research informatics support. J Biomed Inform 42(2):377–381. 10.1016/j.jbi.2008.08.010 PMC2700030 18929686

[CR25] Harris PA, Taylor R, Minor BL, Elliott V, Fernandez M, O’Neal L, McLeod L, Delacqua G, Delacqua F, Kirby J, Duda SN (2019) The REDCap consortium: Building an international community of software platform partners. J Biomed Inform 95:103208. 10.1016/j.jbi.2019.103208 PMC7254481 31078660

[CR27] Hove MJ, Risen JL (2009) It’s All in the Timing: Interpersonal Synchrony Increases Affiliation. Soc Cogn. 10.1521/soco.2009.27.6.949

[CR28] Juslin PN, Västfjäll D (2008) Emotional responses to music: The need to consider underlying mechanisms. Behav Brain Sci 31(5):559–621. 10.1017/S0140525X08005293 18826699

[CR29] Kassambara A, Mundt F (2020) *factoextra: Extract and Visualize the Results of Multivariate Data Analyses* (Version 1.0.7) [Computer software]. https://cran.r-project.org/web/packages/factoextra/index.html

[CR30] Keller PE, Giacomo N, Hove MJ (2014) Rhythm in joint action: Psychological and neurophysiological mechanisms for real-time interpersonal coordination. Philosophical Trans Royal Soc B: Biol Sci 369(1658):20130394. 10.1098/rstb.2013.0394 PMC4240961 25385772

[CR31] Kim YJ, Engel D, Woolley AW, Lin JY-T, McArthur N, Malone TW (2017) What Makes a Strong Team? Using Collective Intelligence to Predict Team Performance in League of Legends. Proc 2017 ACM Conf Comput Supported Coop Work Social Comput CSCW ’17 2316–2329. 10.1145/2998181.2998185

[CR32] Kniffin KM, Yan J, Wansink B, Schulze WD (2017) The sound of cooperation: Musical influences on cooperative behavior. J Organizational Behav 38(3):372–390. 10.1002/job.2128 PMC5347889 28344386

[CR33] Knight AP, Eisenkraft N (2015) Positive is usually good, negative is not always bad: The effects of group affect on social integration and task performance. J Appl Psychol 100(4):1214–1227. 10.1037/apl0000006 25495091

[CR70] Konstabel K, Lönnqvist J-E, Walkowitz G, Konstabel K, Verkasalo M (2012) The ‘Short Five’ (S5): Measuring personality traits using comprehensive single items. Eur J Pers 26(1):13–29. 10.1002/per.813

[CR14] De Kort Y, Ijsselsteijn W, Poels K (2007) Digital Games as Social Presence Technology: Development of the Social Presence in Gaming Questionnaire (SPGQ). In: Journal of Applied Mechanics-transactions of The Asme—J APPL MECH

[CR34] Kulla ST, Sungur H, Sumter SR (2024) The role of social presence in live and recorded concert viewing: Effects on enjoyment and emotional well-being. Computers Hum Behav Rep 14:100409. 10.1016/j.chbr.2024.100409

[CR35] Levinson SC (2016) Turn-taking in Human Communication – Origins and Implications for Language Processing. Trends Cogn Sci 20(1):6–14. 10.1016/j.tics.2015.10.010 26651245

[CR36] Li Y, Chen M, Zhang R, Li X (2021) Experiencing happiness together facilitates dyadic coordination through the enhanced interpersonal neural synchronization. Soc Cognit Affect Neurosci 17(5):447–460. 10.1093/scan/nsab114 PMC9071490 34669963

[CR37] Liljeström S, Juslin PN, Västfjäll D (2013) Experimental evidence of the roles of music choice, social context, and listener personality in emotional reactions to music. Psychol Music 41(5):579–599. 10.1177/0305735612440615

[CR38] Lindeman M, Koirikivi I, Lipsanen J (2018) Pictorial Empathy Test (PET): An easy-to-use method for assessing affective empathic reactions. Eur J Psychol Assess 34:421–431. 10.1027/1015-5759/a000353

[CR39] McDonald B, Böckler A, Kanske P (2022) Soundtrack to the social world: Emotional music enhances empathy, compassion, and prosocial decisions but not theory of mind. Emotion 22(1):19–29. 10.1037/emo0001036 35084909

[CR40] Mehr SA, Krasnow MM, Bryant GA, Hagen EH (2020) Origins of music in credible signaling. Behav Brain Sci. 10.1017/S0140525X20000345 PMC7907251 32843107

[CR41] Miu AC, Vuoskoski JK (2017) The social side of music listening: Empathy and contagion in music-induced emotions. Music and empathy. Routledge/Taylor & Francis Group, pp 124–138. 10.4324/9781315596587-7

[CR71] Müllensiefen D, Gingras B, Musil J, Stewart L (2014) The musicality of non-musicians: An index for assessing musical sophistication in the general population. PLoS ONE 9(2):e89642. 10.1371/journal.pone.0089642 PMC3935919 24586929

[CR42] Novembre G, Mitsopoulos Z, Keller PE (2019) Empathic perspective taking promotes interpersonal coordination through music. Sci Rep 9(1):12255. 10.1038/s41598-019-48556-9 PMC6706439 31439866

[CR43] Okuda S, Runco M, Berger D (1991) Creativity and the Finding and Solving of Real-World Problems. J Psychoeducational Assess - J PSYCHOEDUC ASSESS 9:45–53. 10.1177/073428299100900104

[CR44] Onderdijk KE, Swarbrick D, Van Kerrebroeck B, Mantei M, Vuoskoski JK, Maes P-J, Leman M (2021) Livestream Experiments: The Role of COVID-19, Agency, Presence, and Social Context in Facilitating Social Connectedness. Front Psychol 12. 10.3389/fpsyg.2021.647929 PMC8180877 34108911

[CR45] Park SY, Kaneshiro B (2022) User perspectives on critical factors for collaborative playlists. PLoS ONE 17(1):e0260750. 10.1371/journal.pone.0260750 PMC8730407 34986153

[CR46] Park SY, Lee JH, Laplante A, Hu X, Kaneshiro B (2024) Collaborative Playlists around the World: A Cross–Cultural User Study. Trans Int Soc Music Inf Retr. 10.5334/tismir.169

[CR47] Pels F, Kleinert J (2023) Perspectives on group flow: Existing theoretical approaches and the development of the integrative group flow theory. Group Dynamics: Theory Res Pract 27(4):276–294. 10.1037/gdn0000194

[CR48] Pels F, Kleinert J, Mennigen F (2018) Group flow: A scoping review of definitions, theoretical approaches, measures and findings. PLoS ONE 13(12):e0210117. 10.1371/journal.pone.0210117 PMC6312318 30596773

[CR49] Peñalver J, Salanova M, Martínez IM, Schaufeli WB (2019) Happy-productive groups: How positive affect links to performance through social resources. J Posit Psychol 14(3):377–392. 10.1080/17439760.2017.1402076

[CR50] Phillips-Silver J, Keller P (2012) Searching for Roots of Entrainment and Joint Action in Early Musical Interactions. Front Hum Neurosci. 10.3389/fnhum.2012.00026 PMC3288575 22375113

[CR72] Raven J (1989) The Raven Progressive Matrices: A review of national norming studies and ethnic and socioeconomic variation within the United States. J Educ Meas 26(1):1–16. 10.1111/j.1745-3984.1989.tb00314.x

[CR51] Rentfrow PJ, Gosling SD (2006) Message in a Ballad: The Role of Music Preferences in Interpersonal Perception. Psychol Sci 17(3):236–242. 10.1111/j.1467-9280.2006.01691.x 16507064

[CR52] Savage PE, Loui P, Tarr B, Schachner A, Glowacki L, Mithen S, Fitch WT (2020) Music as a coevolved system for social bonding. Behav Brain Sci 44:e59. 10.1017/S0140525X20000333 32814608

[CR53] Sawyer RK (2006) Group creativity: Musical performance and collaboration. Psychol Music 34(2):148–165. 10.1177/0305735606061850

[CR54] Schumann S, Klein O, Douglas K, Hewstone M (2017) When is computer-mediated intergroup contact most promising? Examining the effect of out-group members’ anonymity on prejudice. Comput Hum Behav 77:198–210. 10.1016/j.chb.2017.08.006

[CR55] Shehata M, Cheng M, Leung A, Tsuchiya N, Wu D-A, Tseng C, Nakauchi S, Shimojo S (2021) Team Flow Is a Unique Brain State Associated with Enhanced Information Integration and Interbrain Synchrony. eNeuro. 10.1523/ENEURO.0133-21.2021 PMC8513532 34607804

[CR56] Stupacher J, Maes P-J, Witte M, Wood G (2017) Music strengthens prosocial effects of interpersonal synchronization – If you move in time with the beat. J Exp Soc Psychol 72:39–44. 10.1016/j.jesp.2017.04.007

[CR57] Stupacher J, Mikkelsen J, Vuust P (2022) Higher empathy is associated with stronger social bonding when moving together with music. Psychol Music 50(5):1511–1526. 10.1177/03057356211050681 PMC9459360 36097608

[CR58] Sutherland ME, Grewe O, Egermann H, Nagel F, Kopiez R, Altenmüller E (2009) The Influence of Social Situations on Music Listening. Ann N Y Acad Sci 1169(1):363–367. 10.1111/j.1749-6632.2009.04764.x 19673809

[CR59] Swarbrick D, Seibt B, Grinspun N, Vuoskoski JK (2021) Corona Concerts: The Effect of Virtual Concert Characteristics on Social Connection and Kama Muta. Front Psychol. https://www.frontiersin.org/articles/10.3389/fpsyg.2021.648448 PMC8260031 34239478

[CR60] Templeton EM, Chang LJ, Reynolds EA, Cone LeBeaumont MD, Wheatley T (2022) Fast response times signal social connection in conversation. Proc Natl Acad Sci 119(4):e2116915119. 10.1073/pnas.2116915119 PMC8794835 35042815

[CR61] Tomprou M, Kim YJ, Chikersal P, Woolley AW, Dabbish LA (2021) Speaking out of turn: How video conferencing reduces vocal synchrony and collective intelligence. PLoS ONE 16(3):e0247655. 10.1371/journal.pone.0247655 PMC7971580 33735258

[CR62] Upham F, Lee JH, Park SY (2024) Audience reconstructed: Social media interaction by BTS fans during live stream concerts. Front Psychol. 10.3389/fpsyg.2024.1214930 PMC11066592 38708012

[CR63] Valdesolo P, DeSteno D (2011) Synchrony and the social tuning of compassion. Emotion 11(2):262–266. 10.1037/a0021302 21500895

[CR64] Vuoskoski J, Clarke E, DeNora T (2016) Music listening evokes implicit affiliation. Psychol Music 0:030573561668028. 10.1177/0305735616680289

[CR65] Wageman R, Hackman JR, Lehman E (2005) Team Diagnostic Survey: Development of an Instrument. J Appl Behav Sci 41(4):373–398. 10.1177/0021886305281984

[CR69] Wakabayashi A, Baron-Cohen S, Wheelwright S, Goldenfeld N, Delaney J, Fine D, Smith R, Weil L (2006) Development of short forms of the Empathy Quotient (EQ-Short) and the Systemizing Quotient (SQ-Short). Pers Indiv Differ 41(5):929–940. 10.1016/j.paid.2006.03.017

[CR66] Wallot S, Mitkidis P, McGraw JJ, Roepstorff A (2016) Beyond Synchrony: Joint Action in a Complex Production Task Reveals Beneficial Effects of Decreased Interpersonal Synchrony. PLoS ONE 11(12):e0168306. 10.1371/journal.pone.0168306 PMC5172585 27997558

[CR67] Wikström V, Falcon M, Martikainen S, Pejoska J, Durall E, Bauters M, Saarikivi K (2021) Heart Rate Sharing at the Workplace. Multimodal Technol Interact. 10.3390/mti5100060

[CR68] Wiltermuth SS, Heath C (2009) Synchrony and Cooperation. Psychological Science. Sage CA. https://journals.sagepub.com/doi/Los Angeles, CA) 10.1111/j.1467-9280.2008.02253.x 19152536

