# Supplementary material for: Shared music listening, emotional arousal and cognitive empathy enhance social presence in online interactions
Source: Cogn Process. 2026 Oct 6;27(4):1489–504. doi: 10.1007/s10339-026-01395-9 (PMC13713997; doi:10.1007/s10339-026-01395-9)
Supplement: Supplementary file 1 [file 10339_2026_1395_MOESM1_ESM.docx]

Supporting Information for

**Shared Music Listening, Emotional Arousal and Cognitive Empathy Enhance Social Presence in Online Interactions**

**Contents**

**Social Presence and Affective Responses During Music Listening**

Table S1. Model Selection for Social Presence and Music-Related Emotions

Table S2. Fixed Effects of Predictors on Social Presence, Music-Induced Arousal, and Music-Induced Valence

Table S3. Random Effects and Model Information for Social Presence, Music-Induced Arousal, and Music-Induced Valence

**Cooperation Following Shared Music Listening**

Table S4. Principal Component Loadings, Explained Variance, and Cumulative Variance for Interaction Quality Items

Table S5. Model Selection for Task Performance

Table S6. Model Selection Results for Group Flow (PC1)

Table S7. Model Selection Results for Emotional Interdependence (PC2)

Table S8. Model Selection Results for Behavioral Interdependence (PC3)

Table S9. Fixed Effects of Predictors on Group Flow, Emotional Interdependence, and Behavioral Interdependence

Table S10. Random Effects and Model Information for Group Flow, Emotional Interdependence, and Behavioral Interdependence

# Social Presence and Affective Responses During Music Listening

Table S1. Model Selection for Social Presence and Music-Related Emotions

| **Outcome** | **Added variable** | **npar** | | **AIC** | | **BIC** | **logLik** | **Deviance** |
| --- | --- | --- | --- | --- | --- | --- | --- | --- |
| Social presence (SPI) |  | |  | |  |  |  |  |
|  | Null model | | 3 | | 440.53 | 449.60 | -217.27 | 434.53 |
|  | Listening repetition (vs. null model) | | 4 | | 440.98 | 453.08 | -216.49 | 432.98 |
|  | Listening condition (vs. null model) | | 4 | | 431.32 | 443.42 | -211.66 | 423.32 |
|  | Preference (vs. listening condition) | | 5 | | 431.73 | 446.86 | -210.87 | 421.73 |
|  | Arousal (vs. listening condition) | | 5 | | 430.86 | 445.98 | -210.43 | 420.86 |
|  | Preference × listening condition (vs. listening condition) | | 6 | | 430.86 | 449.01 | -209.43 | 418.87 |
|  | Valence (vs. Arousal) | | 6 | | 432.85 | 451.00 | -210.43 | 420.85 |
|  | PET (vs. Arousal) | | 6 | | 432.57 | 450.72 | -210.29 | 420.57 |
|  | RMIE (vs. Arousal) | | 6 | | 432.34 | 450.48 | -210.17 | 420.34 |
|  | Arousal × listening condition (vs. Arousal) | | 6 | | 432.42 | 450.57 | -210.21 | 420.42 |
|  | Valence × listening condition (vs. Arousal) | | 7 | | 434.51 | 455.67 | -210.25 | 420.51 |
|  | PET × listening condition (vs. Arousal) | | 7 | | 434.16 | 455.33 | -210.08 | 420.16 |
|  | **RMIE × listening condition (vs. Arousal)** | | **7** | | **406.15** | **427.32** | **-196.07** | **392.15** |
| Music-induced arousal (SAM) |  | |  | |  |  |  |  |
|  | Null model | | 3 | | 671.24 | 680.31 | -332.62 | 665.24 |
|  | Listening repetition (vs. null model) | | 4 | | 673.10 | 685.19 | -332.55 | 665.10 |
|  | Listening condition (vs. null model) | | 4 | | 673.24 | 685.33 | -332.62 | 665.24 |
|  | Preference (vs. Arousal) | | 4 | | 672.64 | 684.74 | -332.32 | 664.64 |
|  | PET (vs. null model) | | 4 | | 671.81 | 683.90 | -331.90 | 663.81 |
|  | RMIE (vs. null model) | | 4 | | 671.45 | 683.55 | -331.73 | 663.45 |
|  | Preference × listening condition (vs. null model) | | 6 | | 675.44 | 693.58 | -331.72 | 663.44 |
|  | PET × listening condition (vs. null model) | | 6 | | 675.80 | 693.95 | -331.90 | 663.80 |
|  | **RMIE × listening condition (vs. null model)** | | **6** | | **669.42** | **687.56** | **-328.71** | **657.42** |
| Music-induced valence (SAM) |  | |  | |  |  |  |  |
|  | Null model | | 3 | | 568.85 | 577.92 | -281.42 | 562.85 |
|  | **Listening repetition (vs. null model)** | | **4** | | **566.80** | **578.90** | **-279.40** | **558.80** |
|  | Listening condition (vs. Listening repetition) | | 5 | | 568.64 | 583.76 | -279.32 | 558.64 |
|  | Preference (vs. Listening repetition) | | 5 | | 566.95 | 582.07 | -278.48 | 556.95 |
|  | PET (vs. Listening repetition) | | 5 | | 568.62 | 583.74 | -279.31 | 558.62 |
|  | RMIE (vs. Listening repetition) | | 5 | | 567.04 | 582.16 | -278.52 | 557.04 |
|  | PET × listening condition (vs. Listening repetition) | | 7 | | 570.45 | 591.62 | -278.23 | 556.45 |
|  | RMIE × listening condition (vs. Listening repetition) | | 7 | | 570.88 | 592.05 | -278.44 | 556.88 |

Note. For each outcome, model selection results are provided, including the number of parameters (npar), Akaike Information Criterion (AIC), Bayesian Information Criterion (BIC), log-likelihood (logLik), and deviance. Models were compared against the null model or the designated reference model. Lower AIC values indicate better model fit.

Table S2. Fixed Effects of Predictors on Social Presence, Music-Induced Arousal, and Music-Induced Valence

| **Outcome** | **Fixed Effects** | **Estimate (SE)** | **95% CI** | **p** |
| --- | --- | --- | --- | --- |
| Social presence (SPI) |  |  |  |  |
|  | **Intercept** | **2.78 (1.30)** | **0.21 – 5.36** | **0.034** |
|  | **Listening condition** | **-2.50 (0.49)** | **-3.47 – -1.52** | **<0.001** |
|  | RMIE score | -0.03 (0.05) | -0.12 – 0.06 | 0.510 |
|  | **SAM arousal** | **0.09 (0.03)** | **0.02 – 0.15** | **0.006** |
|  | **Listening condition × RMIE** | **0.10 (0.02)** | **0.06 – 0.13** | **<0.001** |
| Music-induced arousal (SAM) |  |  |  |  |
|  | **Intercept** | **7.66 (1.54)** | **4.61 – 10.71** | **<0.001** |
|  | **Listening condition** | **3.52 (1.44)** | **0.68 – 6.36** | **0.015** |
|  | RMIE | -0.07 (0.06) | -0.18 – 0.04 | 0.190 |
|  | **Listening condition × RMIE** | **-0.13 (0.05)** | **-0.23 – -0.03** | **0.015** |
| Music-induced valence (SAM) |  |  |  |  |
|  | **Intercept** | **7.79 (0.30)** | **7.19 – 8.39** | **<0.001** |
|  | **Listening repetition** | **-0.22 (0.11)** | **-0.43 – -0.00** | **0.045** |

Note. Fixed effects are reported as estimates with standard errors (SE) and 95% confidence intervals (CI). Outcome variables are social presence (SPI), music-induced arousal (SAM), and music-induced valence (SAM).

Table S3. Random Effects and Model Information for Social Presence, Music-Induced Arousal, and Music-Induced Valence

|  | **Model** | | |
| --- | --- | --- | --- |
| Metric | Social presence (SPI) | Music-induced arousal (SAM) | Music-induced valence (SAM) |
| σ² | 0.49 | 4.38 | 2.22 |
| τ00 | 0.75 | 0.17 | 0.14 |
| ICC | 0.60 | 0.04 | 0.06 |
| N | 38 | 38 | 38 |
| Observations | 152 | 152 | 152 |
| Marginal R² / Conditional R² | 0.126 / 0.654 | 0.049 / 0.085 | 0.025 / 0.081 |

*Note.* The table presents residual variances (σ²), random intercept variances (τ₀₀), intraclass correlation coefficients (ICC), sample sizes (N), number of observations, and model fit indices (Marginal and Conditional R²) for each outcome model.

# Cooperation Following Shared Music Listening

Table S4. Principal Component Loadings, Explained Variance, and Cumulative Variance for Interaction Quality Items

|  | **Principal Components** | | | | | | | | | | |
| --- | --- | --- | --- | --- | --- | --- | --- | --- | --- | --- | --- |
|  | **PC1** | **PC2** | **PC3** | PC4 | PC5 | PC6 | PC7 | PC8 | PC9 | PC10 | PC11 |
| Group flow | **0.37** | -0.12 | -0.10 | -0.12 | 0.35 | 0.14 | -0.21 | 0.14 | -0.07 | -0.52 | 0.58 |
| Social cohesion | **0.35** | -0.10 | -0.20 | 0.07 | 0.16 | 0.13 | 0.15 | 0.57 | 0.59 | 0.17 | -0.24 |
| Co-presence | **0.35** | -0.17 | 0.23 | -0.27 | 0.20 | -0.18 | -0.25 | -0.24 | 0.06 | 0.68 | 0.24 |
| Psychological safety | **0.34** | -0.04 | -0.25 | -0.09 | -0.26 | -0.57 | -0.20 | 0.34 | -0.46 | -0.04 | -0.23 |
| Attention allocation | **0.34** | -0.18 | **0.35** | -0.19 | 0.09 | 0.51 | -0.01 | -0.08 | -0.34 | -0.12 | -0.55 |
| Message understanding | **0.33** | -0.09 | -0.19 | -0.08 | -0.61 | 0.07 | -0.21 | -0.45 | 0.41 | -0.22 | -0.04 |
| Equality | **0.31** | -0.23 | -0.28 | 0.31 | 0.23 | -0.19 | 0.63 | -0.42 | -0.14 | -0.02 | -0.02 |
| Resonance | 0.08 | **0.58** | **-0.33** | -0.66 | 0.06 | 0.10 | 0.31 | -0.08 | -0.04 | 0.06 | 0.00 |
| Emotional interdependence | 0.18 | **0.57** | -0.05 | 0.39 | 0.39 | -0.11 | -0.42 | -0.24 | 0.09 | -0.08 | -0.27 |
| Affective understanding | **0.30** | **0.33** | 0.00 | 0.42 | -0.37 | 0.40 | 0.09 | 0.16 | -0.29 | 0.31 | 0.33 |
| Behavioral interdependence | 0.24 | 0.28 | **0.70** | -0.02 | -0.13 | -0.35 | 0.34 | 0.08 | 0.19 | -0.25 | 0.08 |
|  |  |  |  |  |  |  |  |  |  |  |  |
| Proportion of Variance | **0.50** | 0.14 | 0.08 | 0.06 | 0.05 | 0.04 | 0.04 | 0.03 | 0.03 | 0.02 | 0.02 |
| Cumulative Proportion | **0.50** | **0.64** | **0.72** | 0.78 | 0.83 | 0.87 | 0.91 | 0.94 | 0.97 | 0.98 | 1.00 |

Note. Loadings ≥ |0.30| for PC1-PC3 are bolded. The table reports component loadings, the proportion of variance explained by each component, and the cumulative variance.

Table S5. Model Selection for Task Performance

| **Outcome** | Added Variable | **npar** | AIC | **BIC** | **logLik** | **Deviance** |
| --- | --- | --- | --- | --- | --- | --- |
| Task performance | **Null model** | **6** | **198.39** | **212.38** | **-93.20** | **186.39** |
|  | Listening condition | 7 | 200.30 | 216.61 | -93.15 | 186.30 |
|  | Mean arousal | 7 | 200.32 | 216.64 | -93.16 | 186.32 |
|  | Arousal difference | 7 | 200.14 | 216.45 | -93.07 | 186.14 |
|  | Mean valence | 7 | 200.20 | 216.51 | -93.10 | 186.20 |
|  | Valence difference | 7 | 200.31 | 216.63 | -93.16 | 186.31 |
|  | Mean social presence | 7 | 198.87 | 215.19 | -92.44 | 184.87 |
|  | Mean arousal × listening condition | 9 | 204.13 | 225.10 | -93.06 | 186.13 |
|  | Arousal difference × listening condition | 9 | 202.79 | 223.76 | -92.39 | 184.79 |
|  | Mean valence × listening condition | 9 | 204.11 | 225.09 | -93.05 | 186.11 |
|  | Valence difference × listening condition | 9 | 204.23 | 225.21 | -93.11 | 186.23 |
|  | SPI × listening condition | 9 | 201.17 | 222.14 | -91.58 | 183.17 |

Note. For each model, the number of parameters (npar), Akaike Information Criterion (AIC), Bayesian Information Criterion (BIC), log-likelihood (logLik), and deviance are reported. Models were compared against the null model or a specified reference model. Lower AIC values indicate better model fit.

Table S6. Model Selection Results for Group Flow (PC1)

| **Outcome** | **Added Variable** | **npar** | **AIC** | **BIC** | **logLik** | **Deviance** |
| --- | --- | --- | --- | --- | --- | --- |
| PC1 | Null model | 6 | 238.41 | 252.24 | -113.21 | 226.41 |
|  | Task performance (z-score) (vs. null model) | 7 | 234.83 | 250.96 | -110.41 | 220.83 |
|  | **Listening condition (vs. Task performance (z-score))** | **8** | **231.68** | **250.11** | **-107.84** | **215.68** |
|  | Listening condition × task performance (z-score) (vs. listening condition) | 9 | 233.50 | 254.24 | -107.75 | 215.50 |
|  | Mean arousal (vs. listening condition) | 9 | 233.54 | 254.28 | -107.77 | 215.54 |
|  | Arousal difference (vs. listening condition) | 9 | 231.87 | 252.61 | -106.94 | 213.87 |
|  | Mean valence (vs. listening condition) | 9 | 233.12 | 253.86 | -107.56 | 215.12 |
|  | Valence difference (vs. listening condition) | 9 | 233.52 | 254.25 | -107.76 | 215.52 |
|  | Mean social presence (vs. listening condition) | 9 | 233.56 | 254.30 | -107.78 | 215.56 |
|  | Mean arousal × listening condition (vs. listening condition) | 10 | 235.49 | 258.53 | -107.74 | 215.49 |
|  | Arousal difference × listening condition (vs. listening condition) | 10 | 233.72 | 256.76 | -106.86 | 213.72 |
|  | Mean valence × listening condition (vs. listening condition) | 10 | 235.03 | 258.07 | -107.51 | 215.03 |
|  | Valence difference × listening condition (vs. listening condition) | 10 | 235.18 | 258.22 | -107.59 | 215.18 |
|  | Mean social presence × listening condition (vs. listening condition) | 10 | 235.45 | 258.49 | -107.72 | 215.45 |

Note. For each model, the number of parameters (npar), Akaike Information Criterion (AIC), Bayesian Information Criterion (BIC), log-likelihood (logLik), and deviance are reported. Models were compared against the null model or a specified reference model. Lower AIC values indicate better model fit.

Table S7. Model Selection Results for Emotional Interdependence (PC2)

| **Outcome** | **Added Variable** | **npar** | **AIC** | **BIC** | **logLik** | **Deviance** |
| --- | --- | --- | --- | --- | --- | --- |
| PC2 | Null model | 6 | 174.68 | 188.50 | -81.34 | 162.68 |
|  | Task performance (z-score) (vs. null model) | 7 | 176.29 | 192.42 | -81.14 | 162.29 |
|  | Listening condition (vs. null model) | 7 | 176.12 | 192.25 | -81.06 | 162.12 |
|  | Mean arousal (vs. null model) | 7 | 173.03 | 189.16 | -79.52 | 159.03 |
|  | Arousal difference (vs. mean arousal) | 8 | 168.99 | 187.43 | -76.50 | 152.99 |
|  | Task performance (z-score) × listening condition (vs. null model) | 9 | 179.69 | 200.43 | -80.84 | 161.69 |
|  | Mean arousal × listening condition (vs. mean arousal) | 9 | 176.44 | 197.18 | -79.22 | 158.44 |
|  | **Arousal difference × listening condition (vs. arousal difference)** | **10** | **166.19** | **189.23** | **-73.10** | **146.19** |
|  | Mean social presence (vs. Arousal difference × listening condition) | 10 | 167.10 | 190.14 | -73.55 | 147.10 |
|  | Mean valence (vs. Arousal difference × listening condition) | 11 | 168.17 | 193.51 | -73.08 | 146.17 |
|  | Valence difference (vs. Arousal difference × listening condition) | 11 | 167.95 | 193.30 | -72.98 | 145.95 |
|  | Mean social presence × listening condition (vs. Arousal difference × listening condition) | 11 | 166.86 | 192.21 | -72.43 | 144.86 |
|  | Mean valence × listening condition (vs. Arousal difference × listening condition) | 12 | 170.02 | 197.67 | -73.01 | 146.02 |
|  | Valence difference × listening condition (vs. Arousal difference × listening condition) | 12 | 169.95 | 197.60 | -72.98 | 145.95 |

Note. This table displays the model selection results for PC2. Each model’s performance is evaluated by reporting the number of parameters (npar), AIC, BIC, log-likelihood (logLik), and deviance. Models were compared against the null model or the designated reference model, with lower AIC values indicating better fit.

Table S8. Model Selection Results for Behavioral Interdependence (PC3)

| **Outcome** | **Added Variable** | **npar** | **AIC** | **BIC** | **logLik** | **Deviance** |
| --- | --- | --- | --- | --- | --- | --- |
| PC3 | Null model | 6 | 149.31 | 163.13 | -68.65 | 137.31 |
|  | Task performance (z-score) (vs. null model) | 7 | 145.39 | 161.52 | -65.69 | 131.39 |
|  | Listening condition (vs. Task performance (z-score)) | 8 | 147.36 | 165.79 | -65.68 | 131.36 |
|  | Mean arousal (vs. Task performance (z-score)) | 8 | 147.35 | 165.78 | -65.67 | 131.35 |
|  | Listening condition × task performance (z-score) (vs. Task performance (z-score)) | 9 | 149.35 | 170.08 | -65.67 | 131.35 |
|  | Arousal difference (vs. Task performance (z-score)) | 9 | 148.72 | 169.45 | -65.36 | 130.72 |
|  | Mean valence (vs. Task performance (z-score)) | 9 | 149.04 | 169.78 | -65.52 | 131.04 |
|  | Mean arousal × listening condition (vs. Task performance (z-score)) | 10 | 150.41 | 173.45 | -65.20 | 130.41 |
|  | Arousal difference × listening condition (vs. Task performance (z-score)) | 10 | 146.22 | 169.27 | -63.11 | 126.22 |
|  | Mean valence × listening condition (vs. Task performance (z-score)) | 10 | 135.50 | 158.54 | -57.75 | 115.50 |
|  | Valence difference (vs. mean valence × listening condition) | 11 | 136.88 | 162.23 | -57.44 | 114.88 |
|  | Mean social presence (vs. Mean valence × listening condition) | 11 | 137.31 | 162.66 | -57.66 | 115.31 |
|  | **Valence difference × listening condition (vs Mean valence × listening condition)** | **12** | **136.50** | **164.15** | **-56.25** | **112.50** |
|  | Mean social presence × listening condition (vs. Mean valence × listening condition) | 12 | 139.06 | 166.71 | -57.53 | 115.06 |

Note. This table shows the model selection results for PC3. The table includes the number of parameters (npar), AIC, BIC, log-likelihood (logLik), and deviance for each model. Models were evaluated by comparison with the null model or a designated reference model. Lower AIC values indicate better fit.

Table S9. Fixed Effects of Predictors on Group Flow (PC1), Emotional Interdependence (PC2), and Behavioral Interdependence (PC3)

| **Outcome** | **Fixed Effects** | **Estimate (SE)** | **95% CI** | **p** |
| --- | --- | --- | --- | --- |
| PC1 | Intercept | -0.02 (0.33) | -0.67 – 0.64 | 0.960 |
|  | **Task repetition** | **-0.21 (0.10)** | **-0.41 – -0.01** | **0.037** |
|  | **Task type** | **-0.24 (0.09)** | **-0.42 – -0.06** | **0.011** |
|  | **Task performance** | **0.36 (0.14)** | **0.07 – 0.64** | **0.015** |
|  | **Listening condition** | **-0.20 (0.09)** | **-0.38 – -0.02** | **0.030** |
|  | Task repetition × task type | -0.16 (0.09) | -0.35 – 0.02 | 0.101 |
| PC2 | Intercept | -0.13 (0.34) | -0.81 – 0.54 | 0.692 |
|  | **Task repetition** | **-0.25 (0.06)** | **-0.38 – -0.13** | **<0.001** |
|  | Task type | 0.08 (0.06) | -0.05 – 0.21 | 0.227 |
|  | **Arousal difference** | **-0.12 (0.04)** | **-0.20 – -0.04** | **0.004** |
|  | Mean arousal | 0.07 (0.04) | -0.02 – 0.16 | 0.122 |
|  | **Listening condition × arousal difference** | **-0.10 (0.04)** | **-0.18 – -0.01** | **0.026** |
| PC3 | Intercept | 0.44 (0.44) | -0.43 – 1.32 | 0.316 |
|  | Task repetition | -0.05 (0.06) | -0.17 – 0.07 | 0.430 |
|  | **Task type** | **-0.16 (0.06)** | **-0.27 – -0.05** | **0.005** |
|  | **Task performance** | **-0.22 (0.08)** | **-0.38 – -0.06** | **0.006** |
|  | **Listening condition** | **1.54 (0.39)** | **0.75 – 2.33** | **<0.001** |
|  | **Task repetition × task type** | **0.12 (0.06)** | **0.01 – 0.23** | **0.036** |
|  | Mean valence | -0.06 (0.06) | -0.18 – 0.05 | 0.291 |
|  | **Listening condition × mean valence** | **-0.21 (0.05)** | **-0.32 – -0.11** | **<0.001** |

Note. This table presents the fixed effects of predictors on PC1, PC2, and PC3. Estimates, standard errors (SE), 95% confidence intervals (CI), and p-values are reported.

Table S10. Random Effects and Model Information for Group Flow (PC1), Emotional Interdependence (PC2), and Behavioral Interdependence (PC3)

| **Model** | **PC1** | **PC2** | **PC3** |
| --- | --- | --- | --- |
| σ² | 0.61 | 0.26 | 0.21 |
| τ₀₀ | 1.90 | 0.63 | 0.19 |
| ICC | 0.76 | 0.71 | 0.48 |
| N | 19 | 19 | 19 |
| Observations | 74 | 74 | 74 |
| Marginal R² / Conditional R² | 0.112 / 0.785 | 0.127 / 0.745 | 0.256 / 0.610 |

*Note.* This table presents the residual variances (σ²), random intercept variances (τ₀₀), intraclass correlation coefficients (ICC), sample sizes, total observations and model fit indices (Marginal and Conditional R²) for the three models.
